# Why NIPT should be publicly funded

**DOI:** 10.1136/medethics-2020-106218

**Published:** 2020-04-10

**Authors:** Eline Maria Bunnik, Adriana Kater-Kuipers, Robert-Jan H Galjaard, Inez de Beaufort

**Affiliations:** 1 Medical Ethics, Philosophy and History of Medicine, Erasmus MC, University Medical Centre Rotterdam, Rotterdam, The Netherlands; 2 Clinical Genetics, Erasmus MC, University Medical Centre Rotterdam, Rotterdam, The Netherlands

**Keywords:** autonomy, genetic screening/testing, interests of woman/fetus/father, public policy, reproductive medicine

## Abstract

Asking pregnant women to (co)pay for non-invasive prenatal testing (NIPT) out of pocket leads to unequal access across socioeconomic strata. To avoid these social justice issues, first-trimester prenatal screening should be publicly funded in countries such as the Netherlands, with universal coverage healthcare systems that offer all other antenatal care services and screening programmes free of charge. In this reply, we offer three additional reasons for public funding of NIPT. First, NIPT may not primarily have medical utility for women and children, but rather offers relevant information and reproductive options, and thus serves important autonomy interests of women. Second, public funding of NIPT can be justified because it results in a reduction of collectively borne costs associated with care and support for children with chromosomal abnormalities. It is important to note that this is *not* an argument for individual women to take part in screening or to terminate an affected pregnancy. However, it *is* a legitimate argument in policy making regarding funding arrangements for screening programmes. Finally, public funding would help to amend current misunderstandings among pregnant women (eg, that they are not at risk), and thus to support informed consent for first-trimester prenatal screening.

In a recent paper in *Journal of Medical Ethics*, we have shown that policies requiring pregnant women to (co)pay for non-invasive prenatal testing (NIPT) raise financial barriers that will not improve informed decision making.^1^ Also, they may exacerbate problems regarding equity of access to prenatal screening by disproportionally affecting women of lower socioeconomic status.^1^ In a response, Dagmar Schmitz rightfully points out that the paper does not, however, provide a justification for public funding of NIPT tout court.^2^ Why should NIPT be collectively funded, Schmitz asks, in societies that seem to harbour a ‘growing unease’ with the introduction of NIPT, which is perceived to convey discriminatory messages especially about Down syndrome and is ‘obviously distressing for some’?

First of all, we are not convinced by this version of the expressivist argument; an NIPT programme need not express negative valuations of people with disabilities. On a personal level, for instance, it is perfectly possible to love and cherish a child with a disability and, in a subsequent pregnancy, to make use of a prenatal screening programme aimed at detecting same abnormality and even to terminate an affected pregnancy. On a societal level, likewise, it is not inconsistent to respect and value fellow citizens with disabilities and to contribute (eg, financially, by paying taxes and healthcare premiums) to the provision of care and support for them, and at the same time to approve of prenatal screening programmes. This mirrors an argument by John Harris from 2001 that having a rational preference for one’s children not to be disabled is not the same as ‘having a rational preference for the non-disabled as persons’.^3^

Moreover, prenatal screening does not aim at preventing the birth of people with disabilities in society, but at helping individual pregnant women make important and highly personal reproductive decisions. Admittedly, these decisions are—rightfully—limited to dealing with serious medical conditions, and often result either in termination of pregnancy or in tailoring of prenatal and perinatal care provision to prevent avoidable harm to the child. The programme need not—and should not—express a preference for a certain course of action (ie, termination of pregnancy). However, Bjørn Hofmann, among others, has pointed out that the aggregate of personal decisions about selection might still work to consolidate and reinforce collective norms and values, and, in this case, might lead to a disvaluing of persons with disabilities.^4^ Furthermore, persons with disabilities or their parents may *feel* offended by the existence of screening programmes. We do not mean to deny these concerns. However, they do not trump the importance of making NIPT available to pregnant women to promote reproductive autonomy, and should be mitigated in other ways.

We agree with Schmitz that public funding for NIPT cannot and should not be justified with an appeal solely to its medical utility for women or children. Pregnant women choose to use NIPT not to promote their (mental) health, but because it offers relevant information and reproductive options. Having a child with a chromosomal abnormality undeniably has an impact on the lives of mothers and other family members, which not all mothers are willing or able to take on. The decision to either end an affected pregnancy or carry it to term, is often a very difficult but important life decision. NIPT is justified by its medical utility, and more so by the autonomy interests of pregnant women and their partners. Public funding helps to ensure that NIPT is made available to all pregnant women who wish to make use of it, voluntarily, regardless of their ability to (co)pay. Needless to say, women should be equally free to decline screening.

Second, public funding of NIPT can be justified because it is likely to save the public money. In the Netherlands, an estimated 85% of pregnant women and their partners decide to terminate a pregnancy when trisomy 21 or Down syndrome has been detected and confirmed.^5^ Although a decision to undergo an abortion should not be motived by publicly or privately incurred costs, it does have financial implications for women and for the state. This argument is often the ‘elephant in the room’ in discussions about funding of prenatal screening. In many countries, the costs associated with offering NIPT free of charge to all pregnant women are likely to be less than the costs associated with providing medical care and other forms of care and support to persons with serious congenital disorders and their families. Again, money should not be an argument for individual women to take part in screening or to decline it, or to continue an affected pregnancy or to terminate it. But it can be a legitimate argument in policy making regarding funding arrangements for screening programmes.

Third, public funding may help to improve informed decision making and restore correct understandings of the target group of first-trimester screening programmes in countries like the Netherlands, with universal coverage healthcare systems. As we have argued, funding policies are not neutral:^1^ by offering NIPT free of charge, the state may be seen to support or endorse screening, and, vice versa, by requesting a (co)payment, the state may be seen to discourage screening or deem it unimportant. The latter is of particular concern in our country. In the Netherlands, population screening is generally funded by the state, and antenatal care, which includes prenatal screening for infectious diseases and erythrocyte immunisation and the anomaly scan at 20 weeks of gestation, is fully reimbursed through health insurance. By contrast, women must pay for the combined test out of pocket or copay for NIPT (both approximately €175).^6^ Thus, the (co)payment requirement sets first-trimester screening apart from other antenatal care services, including the 20-week ultrasound scan, which is offered free at the point of care. In addition to other factors,^7^ the (co)payment requirement may explain the limited uptake of first-trimester screening (approximately a third in 2016 with the combined test,^8^ and 46% now that NIPT has been introduced^9^) as compared with the 20-week ultrasound (85%).^8^ It may lead pregnant women to conclude that first-trimester screening is ‘not necessary’ for them.^10^ Although ‘necessity’ (or lack thereof) may not apply to prenatal screening, our point is that in the Netherlands, women may respond to a (co)payment requirement by systematically underestimating the relevance of first-trimester screening, assuming that they are not at risk of having a child with a disability.^10^ Otherwise, the test would have been offered for free. This assumption is false. The risk of being pregnant with a fetus with a trisomy 13, 18 or 21 may be small and may differ among women (for trisomy 21, for instance, it increases with age), but it is present for all women. Schmitz, too, argues that when screening is offered for autonomy interests, all women stand to benefit equally, regardless of a priori risk.

We do believe that NIPT should be offered free of charge to all pregnant women in countries like the Netherlands, which can afford it and have the infrastructure in place to offer it effectively, safely and responsibly.^9^ Public funding of NIPT helps to remove existing inconsistencies between first-trimester prenatal screening and the 20-week ultrasound, to correct misconceptions with regard to the relevance of NIPT for pregnant women, and, importantly, to make NIPT accessible to less privileged women. Also, it will likely be cost-effective. But even if it were not cost-effective, public funding of NIPT can be justified based on the autonomy and social justice interests that it serves.

## References

[1] Bunnik EM, Kater-Kuipers A, Galjaard R-JH, et al Should pregnant women be charged for non-invasive prenatal screening? implications for reproductive autonomy and equal access. J Med Ethics 2020;46(3):194–8. 10.1136/medethics-2019-105675 31527142 PMC7042959

[2] Schmitz D Why public funding for non-invasive prenatal testing (NIPT) might still be wrong: a response to Bunnik and colleagues. J Med Ethics 2020;46:781–2. 31719157 10.1136/medethics-2019-105885

[3] Harris J One principle and three fallacies of disability studies. J Med Ethics 2001;27(6):383–7. 10.1136/jme.27.6.383 11731601 PMC1733465

[4] Hofmann B 'You are inferior!' revisiting the expressivist argument. Bioethics 2017;31(7):505–14. 10.1111/bioe.12365 28614604

[5] de Groot-van der Mooren MD, Tamminga S, Oepkes D, et al Older mothers and increased impact of prenatal screening: stable livebirth prevalence of trisomy 21 in the Netherlands for the period 2000-2013. Eur J Hum Genet 2018;26(2):157–65. 10.1038/s41431-017-0075-1 29330546 PMC5839038

[6] Verweij EJ, Veersema D, Pajkrt E, et al Decision making in prenatal screening: money matters. Acta Obstet Gynecol Scand 2015;94(2):212–4. 10.1111/aogs.12518 25270770

[7] Peters IA, Heetkamp KM, Ursem NTC, et al Ethnicity and language proficiency differences in the provision of and intention to use prenatal screening for Down's syndrome and congenital anomalies. A prospective, non-selected, register-based study in the Netherlands. Matern Child Health J 2018;22(3):343–54. 10.1007/s10995-017-2364-2 28884405 PMC5845051

[8] Liefers J, Cruijsberg J, Harmsen M, et al Monitor 2016: Prenatale screening OP downsyndroom en Het structureel echoscopisch Onderzoek. Nijmegen: Scientific Center for Quality of Healthcare (IQ healthcare), 2017.

[9] van der Meij KRM, Sistermans EA, Macville MVE, et al TRIDENT-2: national implementation of genome-wide non-invasive prenatal testing as a first-tier screening test in the Netherlands. Am J Hum Genet 2019;105(6):1091–101. 10.1016/j.ajhg.2019.10.005 31708118 PMC6904791

[10] Crombag NMTH, Boeije H, Iedema-Kuiper R, et al Reasons for accepting or declining Down syndrome screening in Dutch prospective mothers within the context of national policy and healthcare system characteristics: a qualitative study. BMC Pregnancy Childbirth 2016;16(1):121. 10.1186/s12884-016-0910-3 27229318 PMC4880977

